# Mesenchymal stem cells promote alveolar epithelial cell wound repair in vitro through distinct migratory and paracrine mechanisms

**DOI:** 10.1186/1465-9921-14-9

**Published:** 2013-01-25

**Authors:** Khondoker M Akram, Sohel Samad, Monica A Spiteri, Nicholas R Forsyth

**Affiliations:** 1 Institute for Science and Technology in Medicine, School of Postgraduate Medicine, Keele University, Stoke-on-Trent, Staffordshire ST4 7QB, UK; 2 Department of Respiratory Medicine, University Hospital of North Staffordshire, Stoke-on-Trent, Staffordshire ST4 6QG, UK

**Keywords:** Mesenchymal stem cells, Idiopathic pulmonary fibrosis, Alveolar epithelial wound repair, MSC secretory proteins.

## Abstract

**Background:**

Mesenchymal stem cells (MSC) are in clinical trials for widespread indications including musculoskeletal, neurological, cardiac and haematological disorders. Furthermore, MSC can ameliorate pulmonary fibrosis in animal models although mechanisms of action remain unclear. One emerging concept is that MSCs may have paracrine, rather than a functional, roles in lung injury repair and regeneration.

**Methods:**

To investigate the paracrine role of human MSC (hMSC) on pulmonary epithelial repair, hMSC-conditioned media (CM) and a selected cohort of hMSC-secretory proteins (identified by LC-MS/MS mass spectrometry) were tested on human type II alveolar epithelial cell line A549 cells (AEC) and primary human small airway epithelial cells (SAEC) using an in vitro scratch wound repair model. A 3D direct-contact wound repair model was further developed to assess the migratory properties of hMSC.

**Results:**

We demonstrate that MSC-CM facilitates AEC and SAEC wound repair in serum-dependent and –independent manners respectively via stimulation of cell migration. We also show that the hMSC secretome contains an array of proteins including Fibronectin, Lumican, Periostin, and IGFBP-7; each capable of influencing AEC and SAEC migration and wound repair stimulation. In addition, hMSC also show a strong migratory response to AEC injury as, supported by the observation of rapid and effective AEC wound gap closure by hMSC in the 3D model.

**Conclusion:**

These findings support the notion for clinical application of hMSCs and/or their secretory factors as a pharmacoregenerative modality for the treatment of idiopathic pulmonary fibrosis (IPF) and other fibrotic lung disorders.

## Background

Bone marrow-derived mesenchymal stem cells are a population of multipotent adult stem cells, distinct from haematopoietic stem cells, that classically can differentiate into mesodermal lineages including osteoblasts, chondrocytes, adipocytes, and cardiomyocytes [1-3]. Numerous reports also suggest differentiation into other, non-mesodermal lineages including neurons [4,5], hepatocytes [6] and lung epithelial cells [7-9].

This evidence provides a strong rational for the potential application of hMSCs in regenerative therapeutic approaches in many diseases including those of the lung where effective treatment options may be limited [10]. Idiopathic pulmonary fibrosis (IPF) is a chronic, progressive fibrotic lung disorder of unknown aetiology and the most common and lethal form of interstitial lung diseases with a post diagnosis median survival time of 3–5 years irrespective of its treatment status [11]. Hypothetically, the pathophysiology of IPF is most likely associated with multiple alveolar injuries, failure or delayed alveolar reepithelialisation, abnormal immune responses and subsequent fibrosis [12-14]. Studies involving the bleomycin-induced pulmonary fibrosis mouse model, a widely used animal model of pulmonary fibrosis [15], demonstrated the migration and homing of endotracheal or systematically transplanted MSCs towards the site of injury and attenuation of pulmonary fibrosis [16,17]. However, the magnitude of the amelioration of fibrosis appeared out of proportion to the numbers of engrafted MSCs which had differentiated into alveolar epithelial cells (AECs) indicating the involvement of other mechanisms in this MSC-mediated reparative process. An emerging consensus is that paracrine mechanisms could be associated with MSC-mediated wound repair and tissue regenerative process [18]. However, the identity of these paracrine factors with a putative role in alveolar injury repair and regeneration is not clear.

A wide range of different growth factors, cytokines and extracellular matrix proteins (ECM) have been identified as constituents of the in vitro cultured MSC secretome [19,20]. Many of these secretory proteins are biologically active with anti-inflammatory, anti-fibrotic and immunomodulatory functions [21,22]. Previous reports have demonstrated that conditioned media obtained from MSC culture improved cutaneous wound healing [19,23] and cardiac repair [24]. However, data supporting the role of MSC-secreted paracrine factors in the mediation of AEC wound repair is absent. In this study, we have tested hMSC serum-free conditioned media (CM) on AEC wound repair using an in vitro scratch wound repair assay. We demonstrate that hMSC-CM alone increased AEC migration, contained an array of secretory proteins, but had little impact on the rate of wound repair. However, supplementation of hMSC-CM with trace levels of serum (0.2%) significantly increased both migration and wound repair. A selected cohort of hMSC secretory proteins were tested for their effect on repair of AEC and small airway epithelial cells (SAEC) isolated from small airways of distal human lung, and diverse effects on wound healing and cell migration were noted. By developing a direct contact co-culture wound repair system we also demonstrated that when placed in close proximity, hMSCs would migrate into, and repair, AEC wounds in vitro. These findings provide an insight in understanding the cellular and paracrine effects of hMSC in alveolar wound repair and for possible application of hMSC secretory products or their equivalent recombinant proteins as an alternative pharmacoregenerative therapeutic option for pulmonary fibrosis.

## Methods

### hMSC isolation and cell culture

hMSCs were isolated and expanded from human bone marrow aspirates following previously published methodology [25,26]. Briefly, human whole bone marrow aspirate (collected from iliac crest) (Lonza, USA) was seeded at a density of 10^5^ mononuclear cells/cm^2^ on 10 ng/ml Fibronectin-coated (Cat. No. F0895, Sigma) T-75 tissue culture flasks in 20 ml of DMEM (High glucose, 4.5g/L) supplemented with 5% foetal bovine serum (FBS), 1% L-glutamine, 1% non-essential amino acid (NEAA) and 1% PSA (Penicillin-Streptomycin-Amphotericin B) without any prior gradient centrifugation or immunoselection. Cells were maintained in continuous culture for three weeks in a humidified incubator at 37°C in the presence of 5% CO_2_ and 95% air. After 7 days, half of media was removed and replaced with antibiotic-free medium as described above. Media was replenished after a further week. At the end of third week, the adherent hMSC population was harvested with trypsin and passaged subsequently using 1:4 split ratio for expansion. Passage 1 to 3 cells and their conditioned media were used for all experiments. Human type II alveolar epithelial cell (AEC) line, A549 cells were purchased from ATCC, Rockville, USA and maintained in a continuous culture in DMEM supplemented with 10% FBS, 1% L-glutamine and 1% NEAA and passage 10–45 cells were utilised for wound repair and migration assay. Human primary small airway epithelial cells (SAEC) were purchased from Lonza, USA and cultured following manufacturer instruction using SAGM Bullet Kit (Lonza, USA) complete growth media. SAECs were harvested and passaged using Subculture Reagent Pack (Lonza, USA). Passage 3 to 4 SAECs were used for all experiments. Human normal lung fibroblast cell line CCD-8Lu was purchased from ATCC, Rockville, USA and maintained in a continuous culture (passage 9–14) in DMEM supplemented with 10% FBS, 1% L-glutamine and 1% NEAA. This research did not involve human participants. All cells used in this study were obtained from commercial third party organizations.

### Characterisation of hMSCs and AECs

**Figure 1:**
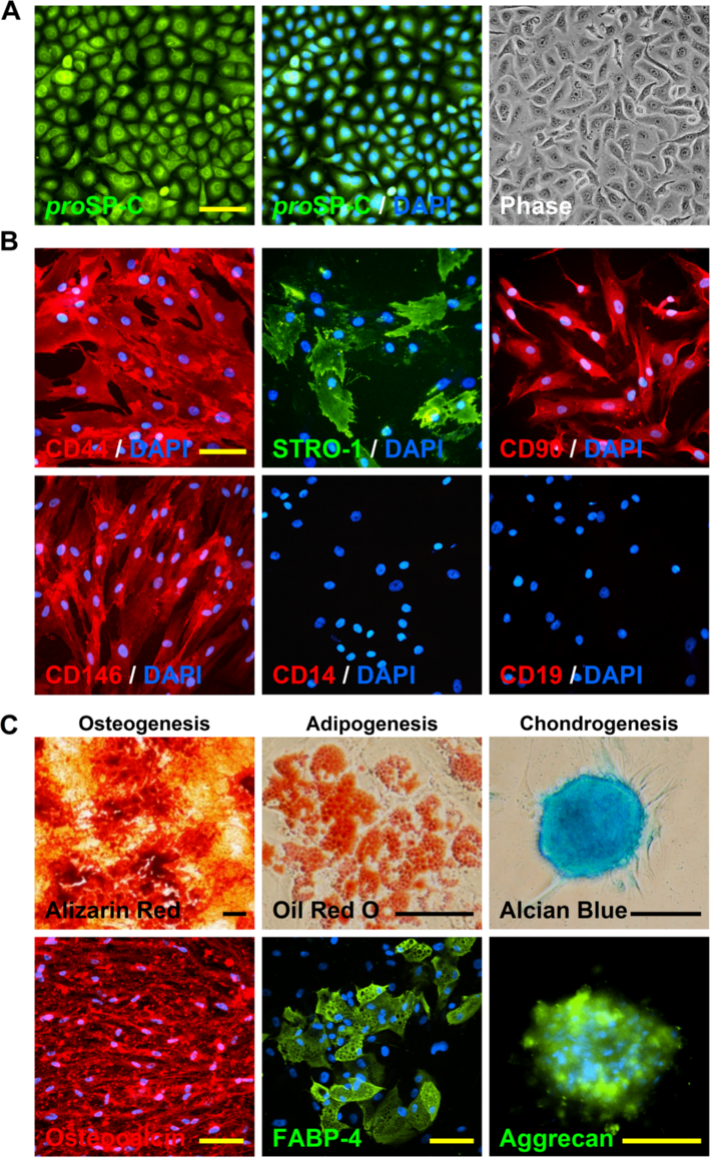
**Characterisation of hMSC and alveolar A549 cells. **(**A**) Human type II alveolar epithelial cell line, A549 cells were positive for type II AEC marker *pro*SP-C. Phase contrast image is taken from an independent field to the *pro*SP-C and *pro*SP-C/DAPI field. (**B**) hMSCs isolated from human bone marrow aspirates were positive for mesenchymal stem cell markers CD44, STRO-1, CD90 and CD146; and negative for haematopoietic markers CD14 and CD19. (**C**) hMSC tri-lineage differentiation: osteogenesis, adipogenesis and chondrogenesis were confirmed by cytochemical/immunocytochemistry staining with Alizarin Red/anti-Osteocalcin, Oil Red O/anti-FABP-4 and Alcian Blue/anti-Aggrecan respectively. Scale bars, 100 μm.

MSCs (passage 1) were plated on 24-well plates and grown to 80-90% confluency for immunophenotyping. Cells were fixed with 4% paraformaldehyde, blocked with 3% BSA (Bovine serum albumin), and characterised using the human MSC characterisation kit containing anti-human mouse anti-CD44, anti-CD90, anti-CD146, anti-CD14, anti-CD19 and anti-STRO-1 primary antibodies at 1:500 dilution (Cat. No. SCR067, Millipore). Cells were incubated with primary antibodies at 4°C overnight. Secondary antibodies were anti-mouse IgG-NL557 for all except STRO-1 and anti-mouse IgM-NL493 for STRO-1 (both at 1:200 dilutions) (R & D System). For functional characterisation, isolated hMSCs were differentiated into osteogenesis, adipogenesis and chondrogenesis lineages using StemPro® hMSC differentiation kits following the manufacturer’s instructions (Gibco, Invitrogen, USA). Briefly, for osteogenesis and adipogenesis, 2 × 10^4^ hMSCs (passage 1) were seeded into each well of 24-well plates and grown to 60-70% confluency in 72 hours using complete MSC culture media as described above. Cells were then cultured for three weeks in StemPro® Osteogenic (Cat. No- A10072-01) and StemPro® Adipogenic (Cat. No- A10070-01) differentiation media with media changes every two days. For chondrogenesis, the micromass culture system was used according to the manufacturer’s instructions. Briefly, 8 × 10^4^ hMSCs (passage 1) were resuspended in 7 μl of complete media and dropped at the centre of a 24-well plate as a micromass and cultured for 2 hours in a humidified incubator in standard culture condition allowing them to adhere with the culture surface. Micromasses were then replenished with StemPro® Chondrogenic differentiation media (Cat. No- A10071-01) and cultured for three weeks in standard culture conditions with media change every two days. Osteogenesis, adipogenesis and chondrogenesis were confirmed by traditional validated cytological staining with Alizarin Red, Oil Red O and Alcian Blue as well as by immunostaining with anti-osteocalcin, anti-FABP-4 and anti-aggrecan antibodies respectively following manufacturer’s protocol (Human MSC Functional Identification Kit, Cat. No- SC006, R & D System). For visualisation, secondary antibodies were used; anti-mouse IgG-NL557 for osteocalcin and anti-goat IgG-NL493 for FABP-4 and aggrecan (both at 1:200 dilutions) (NorthernLights, R & D System). AEC line A549 cells were immunostained with rabbit anti-*pro*SP-C primary antibody (polyclonal, ab40879, Abcam), a specific marker for type II AEC, at 1:250 dilution and FITC-conjugated anti-rabbit secondary was used at 1:200 dilution for visualisation (Abcam). DAPI was used for nuclear staining in all immunocytochemistry assays. Images were acquired by a fluorescent microscope (Nikon Eclipse Ti-ST, Japan) (Figure 1).

### In vitro AEC and SAEC wound repair assay

**Figure 2:**
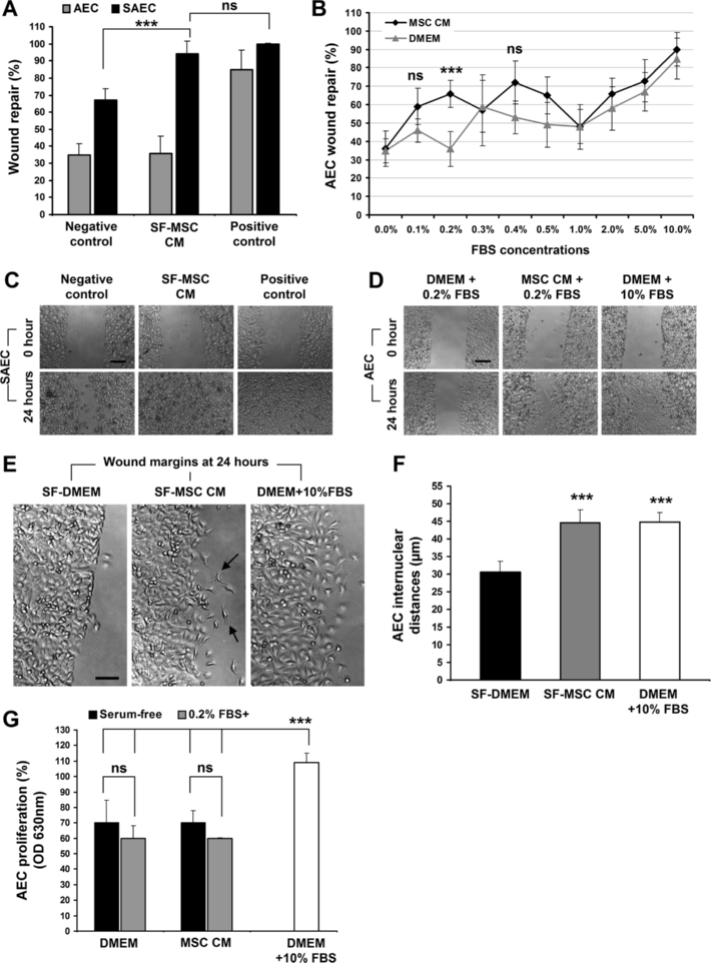
**hMSC paracrine stimulation of AEC and SAEC wound repair in vitro. **(**A**) AEC (grey bars) and SAEC (black bars) wound repair after 24 hours with SF-MSC CM^DMEM^ and SF-MSC CM^SABM^ respectively. Negative controls indicates SF-DMEM for AEC and SF-SABM basal media for SAEC. Positive control represent wound repair with 10% FBS supplemented DMEM for AEC and SAGM for SAEC (AEC, n = 8; SAEC, n = 12; ***p < 0.001). (**B**) AEC wound repair after 24 hours with hMSC conditioned media and DMEM supplemented with different concentration of FBS (n = 8; ***p < 0.001 vs DMEM). (**C**) Inverted light microscopic images of SAEC wound repair. Negative and positive controls indicate serum-free SABM and complete SAGM, respectively. (**D**) Inverted light microscopic images of AEC wound repair. (**E**) Representative inverted light microscopic images of AEC wound margins after 24 hours in SF-DMEM, SF-MSC CM^DMEM^ and 10% FBS supplemented DMEM. Numbers of migrating AECs were observed at the wound margins of SF-MSC CM (arrows) and DMEM + 10% FBS treated samples. (**F**) The internuclear distances of AECs at wound margins after 24 hours; SF-MSC CM^DMEM^ (grey bar), DMEM + 10% FBS (open bar), and SF-DMEM (black bar) (n = 6; ***p < 0.001 vs SF-DMEM). (**G**) AEC proliferation (by MTT assay) after 24 hours of wounding treated with SF (black bars) and 0.2% FBS supplemented (grey bars) DMEM and MSC-CM, and 10% FBS supplemented DMEM (open bar). (n = 4; ***p < 0.001). Data presented as mean ± SD; ns = not significant. Scale bars 200 μm (**C** and **D**) and 100 μm (**E**).

**Figure 3:**
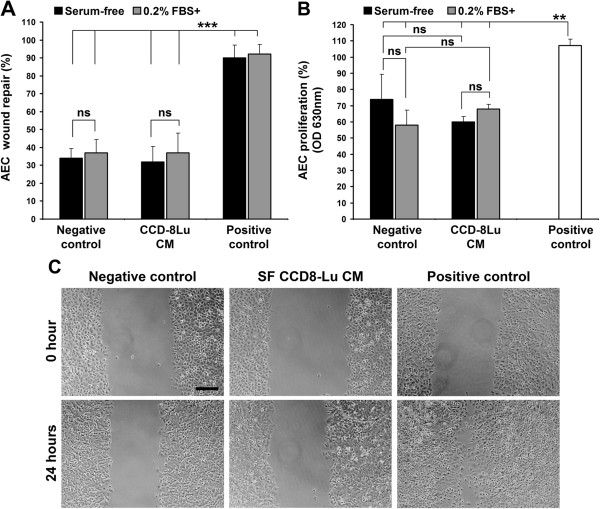
**CCD-8Lu paracrine stimulation of AEC wound repair in vitro. **(**A**) AEC wound repair after 24 hours with SF (black bar) and 0.2% FBS supplemented (grey bar) CCD-8Lu CM. Negative controls represent wound repair with SF-DMEM (black bar) and 0.2% FBS supplemented DMEM (grey bar). Positive control represent wound repair with 10% FBS supplemented DMEM (black bars = positive control for SF-media treated samples; grey bars = positive control for 0.2% FBS supplemented treated samples). (n = 12; ***p < 0.001). (**B**) AEC proliferation (by MTT assay) after 24 hours of wounding treated with SF (black bars) and 0.2% FBS supplemented CCD-8Lu CM (grey bars). Negative controls represent AEC proliferation after 24 hours with SF or 0.2% FBS supplemented DMEM and positive control represents AEC proliferation with 10% FBS supplemented DMEM (open bar). (n = 4; ***p < 0.001). (**C**) Representative inverted light microscopic images of AEC wound repair. Negative control = SF-DMEM, Positive control = 10% FBS supplemented DMEM. Data presented as mean ± SD. ns = not significant. Scale bar, 200 μm.

The in vitro wound repair assay was performed following the protocol described elsewhere [27]. Briefly, AECs and SAEC were cultured to confluence as monolayers on 48-well plates in complete growth media (10% FBS supplemented DMEM for AEC and complete SAGM for SAEC) under standard culture condition. Linear scratch wounds were made on cell monolayers with plastic pipette tips and cell debris were removed by washing with PBS and SF-basal media. Wounded monolayers were then replenished with either serum-free DMEM (SF-DMEM) for AEC and SF-SABM (basal media) for SAEC as negative control, 10% FBS supplemented DMEM for AEC and complete SAGM for SAEC as positive control, serum-free MSC conditioned media (SF-MSC CM^DMEM^ (for AEC) or SF-MSC CM^SABM^ (for SAEC)) or SF-MSC CM^DMEM^ supplemented with different concentrations of FBS for 24 hours (Figure 2A, 2B, 2C and 2D). Wound images were recorded with a digital camera (Canon) attached to an inverted light microscope (Nikon Eclipse, TS100) at 0 and 24 hours (Figure 2C and 2D). Circumferential wound gaps were measured by Image J software (NIH, USA) and percentage of wound repair after 24 hours was calculated. For preparation of MSC-CM, hMSCs (passage 1–3) were grown to 80-90% confluency in T75 tissue culture flasks using 5% FBS supplemented complete MSC culture media. Cells were washed once with PBS and twice with SF-DMEM (or SABM for SAEC) to remove any serum. hMSCs were conditioned by exposing SF-DMEM (for testing on AEC) and SF-SABM (for testing on SAEC) (160 μL/cm^2^) on 80-90% confluent MSCs (8.2 ± 1.2 × 10^5^ cells/flask) for 24 hours in standard culture condition. SF-MSC CM^DMEM^ and SF-MSC CM^SABM^ were collected, centrifuged to remove any cell debris and stored at -80°C and sterile filtered prior to use. Serum-free conditioned media was obtained from 80-90% confluent CCD-8Lu cells by exposing SF-DMEM (160 μL/cm^2^) for 24 hours and stored as described above. In vitro wound repair assay on AEC with CCD-8Lu CM (conditioned media) was replicated as performed using MSC CM described above (Figure 3A, 3C). Serum was added to all CM, as indicated, immediately prior to use. All media was conditioned in a serum-free state.

Internuclear distances between migrated AECs and the cells attached to the wound margins were measured by Image J software. A total 108 measurements were recorded from six individual experiments (Figure 2E and 2F).

### MTT assay

To evaluate the effects of MSC-CM and CCD-8Lu CM on AEC proliferation during wound repair, we have performed a set of MTT (3-(4,5-dimethylthiazol-2-yl)-2,5-diphenyltetrazolium bromide) assay using MTT reagent Thiazolyl Blue Tetrazolium Bromide (Sigma, Cat No- M5655) following manufacturer instructions [28]. Briefly, 10^5^ AEC were grown confluence on each well of 48-well plate in 24 hours and wounds were made as described earlier. For 0 hour reading, MTT was performed just after wounding. Wounded monolayers were washed and 300 μl of 0.5 mg/ml MTT reagent containing SF-DMEM solution was added in each well and incubated at 37°C for 2 hours. After incubation, MTT solution was discarded and washed twice with pre-heated PBS. The formazan was then extracted by 100% DMSO (250 μl/well) incubating 10 minutes in the incubator. 200 μl of extracted formazan-DMSO solution was then loaded in each well of 96-well plate and absorption was measured by micro-plate reader (BioTek, Synergy 2) at 630 nm wavelength. For 24 hours reading, wounded AEC monolayers were treated with SF-MSC CM or 2% FBS supplemented MSC-CM or SF-CCD 8Lu CM or 0.2% FBS supplemented CCD-8Lu CM for 24 hours in the incubator. For negative and positive control, cells were treated with SF-or 0.2% FBS supplemented DMEM and 10% FBS supplemented DMEM respectively. After 24 hours of incubation, media was discarded and MTT was performed as above. Triplicate was done for each sample. Data was presented as percentage increase of optical density (OD) reflecting degree of cell proliferation after 24 hours (Figure 2G, 3B).

### AEC-MSC direct-contact co-culture assay

**Figure 4:**
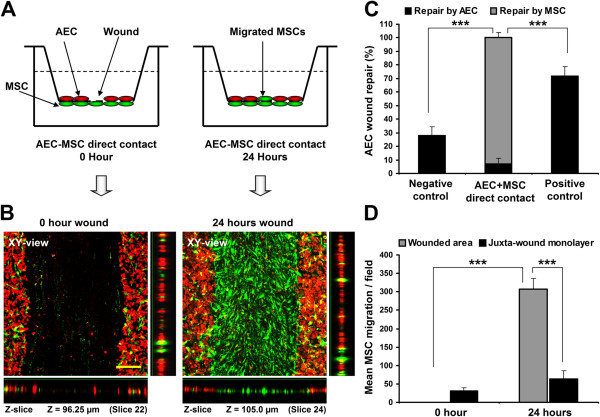
**hMSC migrate to the AEC wound sites and close wound gaps. **(**A**) Schematic diagram showing two-cell direct contact co-culture wound repair model using 3 μm porous transwell system. (**B**) Laser scanning confocal micrographs of AEC-MSC direct contact co-culture wound repair model at 0 and 24 hours. Red cells are DiI labelled AECs and green cells are DiO labelled hMSCs. Horizontal panels are the Z-slicing through the wound gaps and the vertical panels are Z-slicing through the corresponding juxta-wound monolayers. Appearance of green signals in the same plane of red signals in the in the Z-slice after 24 hours confirms hMSC migration to the AEC wound site. (**C**) AEC-MSC direct contact co-culture vs. negative and positive controls. Black and grey bars indicate AEC and hMSC contribution to repair, respectively. Negative control represents AEC wound repair in SF-DMEM in mono-culture setting on upper surface of transwell PET membrane. Positive control represents AEC wound repair in 10% FBS supplemented DMEM in mono-culture setting on upper surface of transwell PET membrane. (n = 6; ***p < 0.001). (**D**) hMSC migration in a direct contact co-culture wound repair system (n = 13; ***p < 0.001). Data presented as mean ± SD. Scale bar, 150 μm.

Direct-contact co-culture wound repair experiment was performed following a previously published methodology with slight modification [29]. Briefly, 10^5^ DiI-labeled AEC and 10^5^ DiO-labeled hMSCs (Vybrant™ Multicolor Cell Labeling Kit, Invitrogen) were grown to confluency in complete culture media on the contra-lateral surfaces of 3 μm porous PET (polyethylene terephthalate) membranes of the 24-well plate format transwell over 24 hours (BD Bioscience) (Figure 4A). Linear wounds were made on AEC monolayers as described above. Wounded cells were then cultured in SF-DMEM for 24 hours (Figure 4B). Prior to imaging hMSCs were removed carefully with a cotton swab from the undersurface of the PET membrane. Cells of upper surface of PET membrane were then fixed with 4% paraformaldehyde. Images were acquired with a laser scanning confocal microscope (Olympus Fluoview) at both 0 and 24 hours. Wound gap measurement and wound repair analysis were performed as described above (Figure 4C).

For assessment of hMSC migration to AEC wound sites in AEC-MSC direct-contact co-culture DiO-labelled hMSCs were counted at the AEC wound gaps and their juxta-wound monolayers at 0 and after 24 hours of wounding (Figure 4D). Confocal Z-scanning was performed to confirm the migration (Figure 4B, vertical and horizontal bars). A minimum of three fields were counted per sample.

### Secretome analysis by mass spectrometry

For identification of secretory proteins in MSC-CM, serum-free and 0.2% FBS supplemented MSC conditioned media was concentrated (Amicon Centriplus Centrifugal Filter Device, 3 kDa MW cut off; Millipore). Samples were reduced and alkylated with 1 μL of 1M DTT for 1 hour at 45°C and 25 μL of 200 mM iodoacetamide for 30 minutes at room temperature in the dark followed by overnight digestion with porcine sequencing grade trypsin (2 μg per sample, Promega). The digested samples were then separated by liquid chromatography. Peptides were eluted for 40 minutes over a 2%-50% MeCN gradient, followed by further elution for 10 minutes at 90% MeCN. Samples were spotted onto a MALDI plate and analyzed in both MS and MS/MS mode on a 4800 MALDI TOF/TOF (Applied Biosystems). Data was searched against the NCBI nr Human database. Proteins that were matched with >95% total ion score C.I% and more than 2 peptides were considered as significant. MSC-CM from three independent donors were evaluated to identify common proteins.

### Testing of proteins on AEC and SAEC wound repair

**Figure 5:**
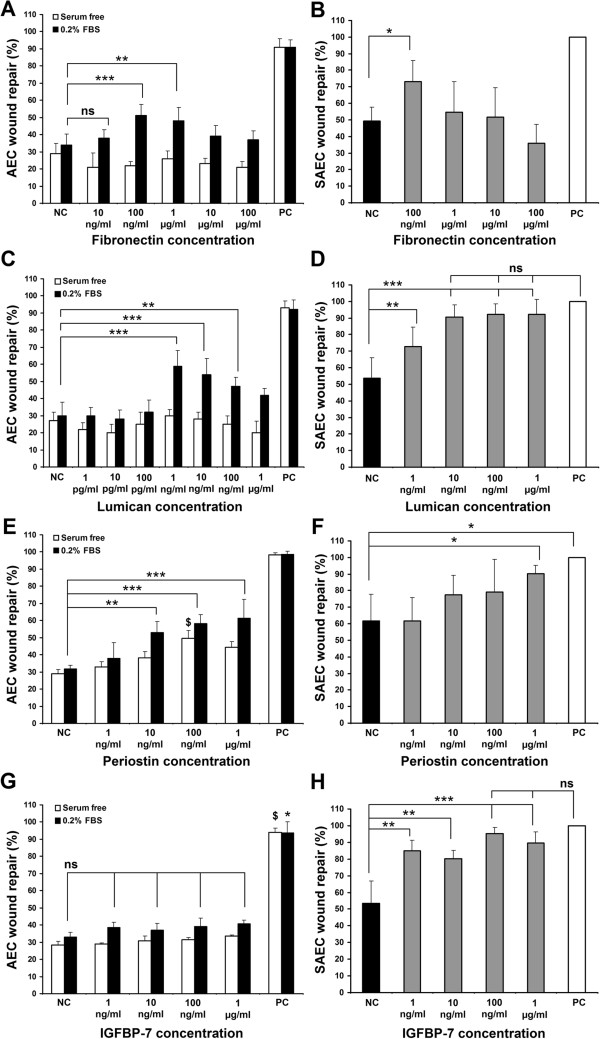
**AEC and SAEC wound repair stimulation by individual hMSC paracrine factors. **(**A**) Recombinant human plasma Fibronectin did not enhance AEC wound in serum-free condition (open bars) but required 0.2% FBS supplementation (black bars) (n = 6; **p < 0.01, ***p < 0.001) (**B**) Recombinant human plasma Fibronectin significantly increased SAEC wound repair at 100 ng/ml concentration in SF-SABM (grey bar) vs. negative control (NC) (black bar) (n = 6; *p < 0.05). (**C**) Recombinant human Lumican did not enhance AEC wound in serum-free condition (open bars) but required 0.2% FBS supplementation (black bars) (n = 9; **p < 0.01, ***p < 0.001). (**D**) Recombinant human Lumican significantly increased SAEC wound repair (n = 6; **p < 0.01, ***p < 0.001). (**E**) Recombinant human Periostin enhanced AEC wound repair (n = 10, **p < 0.01, ***p < 0.001; ^$^p < 0.001 vs corresponding serum-free NC). (**F**) Periostin increased SAEC wound repair at 1 μg/ml in SF-SABM (n = 6; *p < 0.05). (**G**) Human recombinant IGFBP-7 did not stimulate AEC wound repair in serum-free (open bars) or 0.2% FBS supplemented conditions (black bars) (n = 9; *p < 0.001 vs 0.2% FBS supplemented NC, ^$^p < 0.001 vs serum-free NC). (**H**) Recombinant human IGFBP-7 significantly stimulated SAEC wound repair (n = 6; **p < 0.01, ***p < 0.001). NC (negative control) represents wound repair with DMEM for AEC and serum-free SABM for SAEC. PC (positive control) represents wound repair with 10% FBS supplemented DMEM for AEC and complete SAGM for SAEC. Data presented as mean ± SD. ns = not significant. Scale bar, 200 μm.

Plasma Fibronectin (Sigma), Lumican, Periostin and IGFBP-7 (R & D System) recombinant human proteins were assessed in the above described AEC and SAEC in vitro wound repair system. Linear wounds were made on AEC confluent monolayers and treated with the above described proteins in soluble form in SF-DMEM or in 0.2% FBS supplemented DMEM at different concentrations (1 pg/ml to 100 μg/ml). SAEC wounds were treated with above mentioned proteins in SF-SABM for 24 hours. For negative control (NC), wounded AEC were treated with SF-DMEM or 0.2% FBS supplemented DMEM and SAEC were treated with supplement-free basal SABM for 24 hours. For positive control (PC), wounded AEC were treated with 10% FBS supplemented DMEM and SAEC were treated with complete SAGM. Individual positive controls were run for SF and 0.2% FBS supplemented AEC samples. PC were run side-by-side each NC to ascertain the wound repair system was working properly. Percentage of wound repair after 24 hours was calculated as described previously (Figure 5).

### Collagen drop cell migration assay

**Figure 6:**
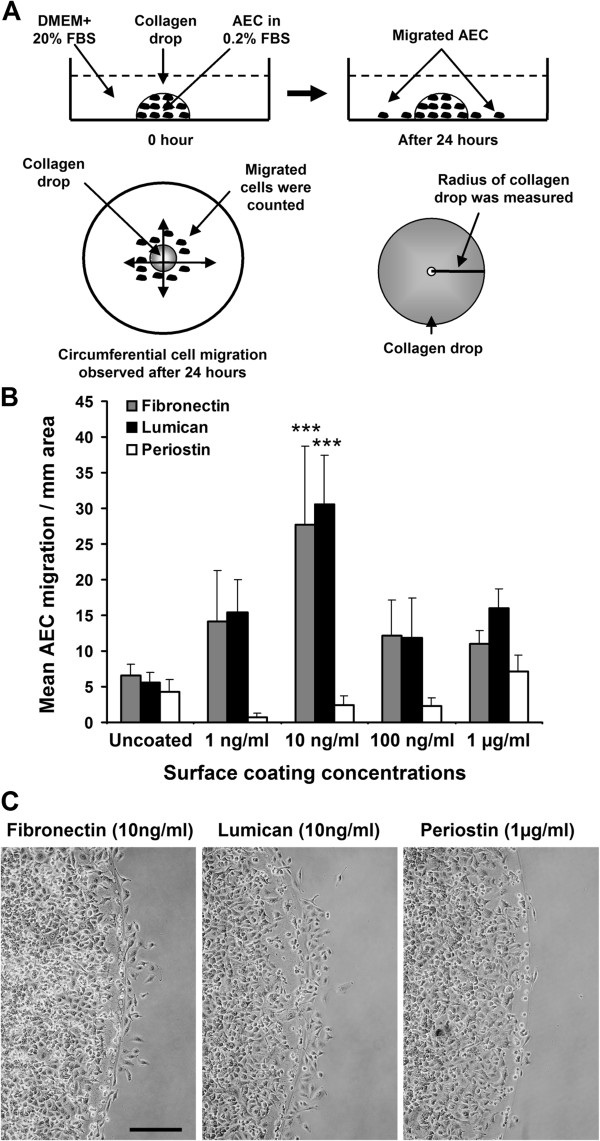
**Fibronectin and Lumican but not Periostin stimulate AEC migration as substrate components. **(**A**) Schematic diagram shows collagen drop cell migration assay. (**B**) Fibronectin and Lumican coating at various concentration stimulate AEC migration (Fibronectin, n = 3; Lumican, n = 6). Periostin coating did not stimulate AEC migration (n = 6). (**C**) Representative images of AECs migration across the collagen-drop barrier. Data presented as mean ± SD. ***p < 0.001. Scale bar, 250 μm.

To evaluate the effect of above identified proteins on AEC migration as substrate components, we developed a cell migration assay tool where 5 × 10^4^ AECs were resuspended in 10 μl of 1 mg/ml concentration of rat tail collagen I (BD Bioscience) in 0.2% FBS supplemented DMEM. The cell/collagen solution was dropped on different concentration of Fibronectin or Lumican or Periostin coated 24-well plates. Well-plates were coated with proteins by adding 500 μl of different concentrations of proteins and incubating them at 4°C over night. After incubation, solution was discarded and wells were air dried before plating the collagen drop. Acidity of collagen was neutralised using 1M NaOH buffer. A single drop (10 μl) was placed at the centre of each well and incubated for 30 minutes in the humidified incubator at 37°C allowing the drops to form into gel. Following gel formation, 600 μL of 20% FBS supplemented DMEM was added to each well to provide a chemoattractant-bias for the promotion of cell migration out of the gel barrier (Figure 6A). After 24 hours migratory cells were quantified microscopically. The radius of each drop was measured by Image J software and the circumference of each drop was calculated. Results are expressed as mean migration through per millimeter of circumference of collagen-drop barrier (Figure 6B, 6C).

### Statistical analysis

The significance of difference between groups was determined by one-way ANOVA with Post-hoc Tukey’s Multiple Comparison analysis. A ‘p’ value less than 0.05 was considered to indicate statistical significant. Data are presented as mean ± standard deviation (SD). All statistical analysis was performed using GraphPad Prism version 5.00 software for Windows (GraphPad Software, San Diego, California, USA).

## Results

### hMSC secretome stimulates alveolar epithelial cell wound repair in a serum-dependant manner

To investigate the role of the hMSC secretome on AEC and SAEC wound repair, human MSC-derived serum-free conditioned media was tested on *pro*Surfactant protein-C (*pro*SP-C) expressing human type II AEC line A549 (AEC) (Figure 1A) and primary human SAEC utilising an established in vitro scratch wound repair model [27]. A characteristic of idiopathic pulmonary fibrosis is aberrant repair processes in wounded alveolar epithelium resulting in denuded epithelium [13]. The source of these wounds remains unknown but disrupted repair of the alveolar epithelium is the underlying principle. The use of the scratch wound repair model replicates the wounds, and through the controlled experimentation we describe, allows replication of the inability to repair and a platform to test novel factors in repair stimulation. hMSC were isolated from bone marrow aspirates according to previously described methodology [25,26] and immunophenotype (CD44^+^, CD90^+^, CD146^+^, STRO-1^+^, CD14^-^, and CD19^-^) (Figure 1B) was confirmed prior to experimental use. The multipotency of hMSC was confirmed by differentiation into osteogenesis, adipogenesis and chondrogenesis lineages, confirmed by use of traditional cytological and immunological studies using Alizarin Red/Osteocalcin, Oil Red O/FABP-4 and Alcian Blue/Aggrecan respectively (Figure 1C). hMSC serum-free conditioned media (SF-MSC CM^DMEM^) did not show any stimulatory effect on AEC wound repair after 24 hours (36.0 ± 9.8% SF-MSC CM^DMEM^ vs. 34.6 ± 6.6% SF-DMEM (negative control)) whereas SAEC wound repair was increased with SF-MSC CM^SABM^ (93.5 ± 7.6% SF-MSC CM^SABM^ vs. 66.8 ± 6.6% SF-SABM (negative control); p < 0.001) (Figure 2A, 2C). Surprisingly, supplementation of SF-MSC CM^DMEM^ with trace amounts of FBS improved the rate of AEC wound repair stimulation when compared with corresponding DMEM controls supplemented with same amounts of FBS (Figure 2B). Maximal effect was observed with the addition of 0.2% FBS in SF-MSC CM^DMEM^ over its corresponding control (0.2% FBS supplemented DMEM) (66.3 ± 7.4% vs. 35.7 ± 9.5%; p < 0.001) (Figures 2B and 2D). The correlation between wound repair rate and increase in FBS addition was poor, though consistent, when FBS concentrations of <1% were examined. This was noted for both SF-MSC CM^DMEM^ and DMEM (Figure 2B).

SF-MSC CM^DMEM^ without additional FBS supplementation did not increase the AEC wound repair rate. However, when exposed to SF-MSC CM^DMEM^ for 24 hours, isolated AECs migrated from their wound margins towards wound gaps with an accompanying migratory morphology (Figure 2E). Inter-nuclear distances, a measure of migration [27,30], in SF-MSC CM^DMEM^ treated AEC were significantly higher than that observed in SF-DMEM treated wounded AECs (44.6 ± 3.7 μm vs. 30.6 ± 3.0 μm; p < 0.001) (Figure 2F). Next, we investigated the influences of 0.2% FBS supplementation with SF-MSC CM^DMEM^ or SF-DMEM on AEC proliferation during in vitro wound repair. The MTT assay, an established method for determination of cell proliferation, demonstrated that supplementation of SF-MSC CM^DMEM^ or SF-DMEM with 0.2% FBS did not increase AEC proliferation after 24 hours of wound repair; whereas, supplementation of 10% FBS with DMEM significantly increased AEC proliferation in comparison to both SF-DMEM and SF-MSC CM^DMEM^ (p < 0.001) (Figure 2G). Taken together, serum-free MSC conditioned media alone can stimulate migratory behaviour of AEC during attempted wound repair but does not induce AEC wound repair without supplementation of additional trace levels of serum. However, SAEC wound repair required SF-MSC CM^SABM^ only. These findings demonstrate a phenotype-dependant and anatomical compartment-specific diverse paracrine response to hMSC secretome on distal airway and AEC wound repair where, at least for the AEC, the reparative process was accomplished by stimulation of cell migration. On the contrary, serum-free or 0.2% FBS supplemented conditioned media obtained from normal human lung fibroblasts CCD-8Lu (CCD-8Lu CM) neither enhanced AEC wound repair (Figure 3A, 3C) nor stimulated AEC cell proliferation (Figure 3B). This observation suggests a distinct influence of hMSC and normal human lung fibroblasts on alveolar epithelial wound repair.

### hMSC migrate into wounded AEC layers in response to injury

hMSC migration to the site of injury is proposed as an important element of AEC wound repair and regeneration in animal models of pulmonary fibrosis [17]. To investigate the migratory properties of hMSC we developed a two-cell co-culture 3D wound repair model (Figure 4A). In response to AEC injury hMSCs migrated into the AEC wound and completely closed the wound gap (Figure 4B and 4C). AEC wound repair was significantly lower in both the negative and positive control than in the AEC-MSC direct contact co-culture (28.0 ± 6.5% (negative control, AEC upper PET membrane surface mono-culture in SF-DMEM), 72.0 ± 6.9% (positive control, AEC upper PET membrane surface mono-culture in 10% FBS DMEM) and 100% (AEC-MSC direct-contact in SF-DMEM) respectively; p < 0.001 vs AEC-MSC direct contact) (Figure 4C). Cell migration assessment demonstrated a 5-fold higher hMSC migration into wound gaps over the juxta-wound monolayers after 24 hours of direct contact co-culture (mean values 308.3 ± 13.9 (wound gap) vs. 66.0 ± 13.4 (juxta-wound margin); p < 0.001) (Figure 4D). In the absence of AEC, hMSC did not migrate to the opposite surface of the porous transwell membrane (data not shown).

### Proteins detected in MSC conditioned media show differential wound repair potential

hMSC conditioned media enhanced AEC migration and wound repair (with trace FBS supplementation). We hypothesised that candidate molecules within the hMSC secretome could enhance alveolar epithelial cell wound repair. To test this hypothesis, we first evaluated the composition of the SF-MSC CM^DMEM^ secretome from hMSC isolated from three donor independent marrow samples using LC-MS/MS mass spectrometry (Table 1). Our primary target cell type was AEC; however, we replicated equivalent experiments on SAEC to compare and contrast the wound repair responses to the paracrine stimuli of commonly identified hMSC-secreted candidate proteins. Fibronectin and Lumican displayed no effect on AEC wound repair rate when added to serum-free medium; however, when supplemented with 0.2% FBS they significantly increased AEC wound repair (Figure 5A and 5C). Fibronectin-induced wound repair was maximal at a concentration of 100 ng/ml when compared to the corresponding control medium (50.8 ± 6.7% vs. 33.8 ± 6.3% (0.2% FBS supplemented DMEM); p < 0.001) (Figure 5A). Lumican displayed maximal wound repair effects at a 1 ng/ml concentration (58.7 ± 8.9% vs. 29.8 ± 3.5% (0.2% FBS supplemented DMEM)); p < 0.001) (Figure 5C). In contrast, Fibronectin and Lumican significantly increased SAEC wound repair without an additional serum supplementation requirement (Figure 5B and 5D). Fibronectin displayed a maximal wound repair effect on SAEC with a 100 ng/ml concentration (73.0 ± 12.9% vs. 49.1 ± 8.5% (SABM); p < 0.05) (Figure 5B); whereas, Lumican displayed significant wound repair effect at a concentration of 100 ng/ml (92.1 ± 6.6% vs. 53.8 ± 12.4% (SABM); p < 0.001) (Figure 5D). Unlike Fibronectin and Lumican, Periostin significantly enhanced AEC wound repair without any additional supplementation where a maximal effect was observed with a 100 ng/ml concentration in serum-free medium (50.0 ± 4.5% vs. 28.9 ± 2.4% (SF-DMEM); p < 0.01) (Figure 5E). Periostin also enhanced SAEC wound repair in serum-free basal medium (90.1 ± 5.2% vs. 61.8 ± 15.9% (SABM); p < 0.05) (Figure 5F). On the other hand IGFBP-7 (Insulin-like growth factor binding protein-7) showed no significant effect on AEC wound repair (Figure 5G); whereas, SAEC wound repair was significantly stimulated by all tested concentrations of IGFBP-7 in serum-free basal media where a maximum effect was observed with 100 ng/ml concentration (95.3 ± 3.5% vs. 53.3 ± 13.3% (SABM); p < 0.001) (Figure 5H). Gelatinase A (MMP2) had no discernable effect on either wound repair rate or migration in AEC wound repair (data not shown).

**Table 1:** Protein components of SF-MSC CM detected by LC-MS/MS mass spectrometry

**Protein name**	**Accession number**	**Average Peptide count**
**Fibronectin** 1, isoform CRA_j	**gi|119590945**	**39.0**
Collagen alpha-2(I) chain	gi|124056488	30.0
Collagen alpha 1 chain precursor variant	gi|62088774	23.5
Pro alpha 1(I) collagen	gi|186893270	23.5
Alpha 1 (I) chain propeptide	gi|180392	21.3
Collagen, type VI, alpha 1 precursor	gi|87196339	11.0
Precursor polypeptide (AA −31 to 1139)	gi|37465	7.5
Actin, gamma 1 propeptide	gi|4501887	7.0
**Gelatinase A**	**gi|5822007**	**6.7**
Keratin 1	gi|7331218	6.5
Tumor necrosis factor	gi|339992	6.3
**Osteoblast specific factor 2 (Periostin)**	**gi|393319**	**6.0**
Chain A, Crystal Structure Of The Thrombospondin-1	gi|88191917	5.5
Biglycan	gi|179433	4.7
**Lumican**	**gi|642534**	**4.7**
Collagen alpha 1(V) chain precursor	gi|219510	4.5
**Insulin-like growth factor binding protein 7, isoform CRA_a**	**gi|119625925**	**4.5**
CALU	gi|49456627	4.0
Thrombospondin 2 precursor	gi|40317628	4.0
Fructose-bisphosphate aldolase A	gi|4557305	3.5
Heparan sulfate proteoglycan	gi|184427	3.5
Pigment epithelium-derived factor	gi|1144299	3.5
Profilin Binds Proline	gi|5822002	3.0
Protein disulfide isomerase	gi|860986	3.0
Versican isoform 3 precursor	gi|255918077	3.0
Decorin	gi|181519	2.7
The Antigenic Identity Of Peptide(Slash)mhc Complexes	gi|442989	2.5
COL1A1 and PDGFB fusion transcript	gi|3288487	2.5
Peptidylprolyl isomerase A	gi|10863927	2.5
Fibrillin	gi|306746	2.0
Similar to cardiac leiomodin	gi|51095092	2.0
Solution Structure Of Calcium-Calmodulin N-Terminal Domain	gi|16974825	1.7
Myosin, light chain 6, alkali, smooth muscle and non-muscle isoform 1	gi|17986258	1.5
Protein disulfide-isomerase A3	gi|729433	1.5
Insulin-like growth factor binding protein 6	gi|183894	1.0

SF-MSC CM preparation was as described in Materials and Methods. Proteins selected for investigations in wound repair and migration are indicated in bold. Accession numbers are derived from MS/MS data searches against non redundant Human NCBI Protein Database. Average peptide count from 3 biologically independent replicates of hMSC secretome profiles.

### Fibronectin and Lumican but not Periostin stimulate AEC migration as substrate components

Fibronectin, Lumican and Periostin induced AEC wound repair when delivered as a culture medium supplement. As these proteins are abundant within the ECM, we hypothesised that these proteins could influence AEC migration as substrate components. Immediate relevance to fibrotic lung disease is evidenced by the abrogation of effective AEC migration over denuded alveolar epithelium. To test this hypothesis, we developed a ‘collagen drop’ cell migration assay where an aggregated population of AECs were resuspended in a drop of collagen which was ‘dropped’ onto a surface coated with Fibronectin, Lumican, or Periostin. To provide an additional ‘positive’ bias to AEC migration the collagen drop contained 0.2% FBS whereas the external serum concentration was 20% (Figure 6A). Optimal cell migration was observed on either Fibronectin or Lumican coated surfaces at a concentration of 10 ng/ml (Fibronectin 27.7 ± 11.1 cells/mm vs. 6.7 ± 1.5 cells/mm (uncoated surface), p < 0.001; Lumican 30.7 ± 6.8 cells/mm vs. 5.7 ± 1.4 cells/mm (uncoated surface), p < 0.001) (Figure 6B, 6C). On the other hand, Periostin did not stimulate AEC migration (Figure 6B, 6C). Taken together this indicates that Fibronectin and Lumican stimulated AEC wound repair as both soluble factors and as a substrate component whereas Periostin stimulated AEC wound repair as a soluble factor only.

## Discussion

Human mesenchymal stem cells (hMSC) are in current clinical trials for incurable diseases including osteogenesis imperfecta, graft-versus-host disease, chronic ischemic heart disease, and chronic obstructive pulmonary disease ( http://www.clinicaltrials.gov) and are the focus of many other clinical applications. Studies on animal pulmonary fibrosis models demonstrate that intravenous and endotracheal administration of MSCs attenuate lung injury and fibrosis suggesting a potential clinical application of MSCs for the treatment of IPF [16,17,31]. However, the mechanism of MSC-mediated amelioration of pulmonary fibrosis is not clear and an active participation of MSCs through differentiation into AEC and lung regeneration is under debate [16,32]. Bleomycin-induced mouse lung fibrosis models have demonstrated an MSC stimulated reduction in pulmonary fibrosis via inhibition of pro-fibrotic cytokines TNF-α and IL-1 through a paracrine mechanism [33]. Furthermore, rat models of pulmonary emphysema demonstrated that MSCs reduce AEC apoptosis through paracrine mechanism via up-regulation of anti-apoptotic Bcl-2 gene [34]. Here we demonstrate that in response to injury, hMSC display site-specific migration into AEC wounds; this observation has also been noted by others in animal lung injury models [16,17]. Coupled to this hMSC migration, we also demonstrate that in response to SF-MSC CM, AEC and SAEC migration and wound repair occur with distinct trace serum augmentation requirements. We also provide evidence supporting specific hMSC-secreted paracrine components for effective alveolar and small airway epithelial wound repair.

hMSC secret a myriad of proteins that include growth factors, cytokines and ECM proteins [19,20]. Many of these secretory proteins are biologically active with anti-inflammatory, anti-fibrotic and immunomodulatory functions [21,22]. Previous and current studies are mostly focused on evaluation of MSC-secreted growth factors and their effects on tissue repair. Here, in line with others, our mass spectrometry-mediated hMSC secretome analyses indicate the presence of a high abundance of ECM/matricellular protein components with diverse biological activities on wound healing and tissue repair [20,23]. Fibronectin (a multifunctional glycoprotein), Lumican (collagen-binding keratin sulfate proteoglycan), Periostin (matricellular N-glycoprotein), and IGFBP-7 (IGF-I, -II low affinity binding protein) were identified as major components of SF-MSC CM through high peptide counts and ion score confidence intervals (Table 1). Data supportive of a role for these factors in alveolar wound repair is relatively scarce though implied in previous studies. Fibronectin upregulation is implicated in abdominal wall, corneal, and skin in vivo and ex vivo wound repair and migration where values ranging from 100 ng/ml to 60 μg/ml of Fibronectin have been evaluated [35-39]. In vitro models including corneal epithelial cells, corneal fibroblasts, keratinocyte, dermal fibroblast, nasal airway epithelial cells, gingival fibroblast, and SAEC have further demonstrated that fibronectin stimulated wound repair and cell migration as either soluble factor or substrate component frequently through integrin signaling activation [40-45].

Lumican, a small leucine-rich proteoglycan (SLRP) family member, is a major component of the proteoglycan-based ECM. Lumican displays a heterogeneous, diffuse, staining profile in the alveolar walls and peripheral regions of adult human lung and its sub-epithelial deposition is implicated in airway remodeling and counteracting the severity of asthma [46,47]. In vivo and ex vivo studies indicate that Lumican is essential for corneal epithelial cell, skin, and oral mucosa wound repair and promotion of chemotactic migration [48-51]. In vitro migration and wound healing of corneal epithelial cells was also induced by Lumican [52,53]. Circulating plasma and cellular Fibronectin concentrations (300 μg/ml and 2.46 μg/ml, respectively) are substantially in excess of those required to induce wound repair and migration in our, and previously described (see above), in vitro models whereas physiological levels of Lumican are yet to be defined [54]. In support and extension of these previous reports we have now demonstrated that both Fibronectin and Lumican facilitated AEC and SAEC wound repair as soluble factors (with trace serum supplementation for AEC only) and migration of AEC as a substrate component.

Periostin (or OSF-2, Osteoblast Specific Factor-2) is a matricellular protein with a poorly defined role in wound repair and a physiological serum level of approximately 39 ng/ml [55,56]. In vivo studies revealed an association with fracture healing, wound-derived blood vessels, acute myocardial infarction response, skin wounds, and ligament repair [57-62]. In addition to those above low levels of Periostin expression are common in normal lung while high levels of Periostin are detected in IPF lungs and patient serum although the role of this protein in the pathogenesis of lung fibrosis has not been clarified [56]. A relationship between Periostin and wound repair across multiple tissues is immediately apparent though little is known, at this time, of mechanism of action. A solitary report describes exogenous overexpression of Periostin in A549 cells, as used in our study, which enhanced both proliferation and migration in routine culture [63]. We have herein reported that Periostin has a role in AEC and SAEC wound repair and migration as a soluble factor and identified a putative hMSC source of wound associated Periostin.

IGFBP-7 (also known as IGFBP-rP1) is an IGFBP-related protein (IGFBP-rPs) superfamily member. IGFBP-rPs have less binding affinity to IGF (insulin growth factor) and are involved in diverse biological activities in an IGF-independent manner [64]. IGFBP-rP2 (CTGF (connective tissue growth factor)), another IGFBP-rP family member, plays a critical role during fibrogenesis in IPF [65,66]. However, the role of IGFBP-7, which has a normal serum concentration of 33 ng/ml [67], in pulmonary fibrosis is unknown. IGFBP-7 is up-regulated in the fibrotic regions of IPF lung tissue as well as in isolated IPF fibroblasts though absent from controls [68]. Here we have demonstrated that recombinant human IGFBP-7 significantly increased human primary SAEC wound repair when applied in serum-free basal media, whereas AEC were non-responsive to this protein. The mechanism behind these divergent responses remains to be clarified. The development of transferable and accessible primary human AEC cultures (which replicate in vivo characteristics; i.e. monolayer formation) will be necessary before clarification can be achieved. However at this time we cannot preclude the possibility that the distinct response is due tissue source or phenotypic background of the AEC and SAEC used in this study. However this distinction in response provides strong support for broad therapeutic applicability across multiple clinical applications for the hMSC secretome. Characterization of these cell-specific responses will underpin the continuing development of the stem cell-driven regenerative medicine industry.

The protein composition of ECM is variable, tissue specific, and provides essential scaffold and biochemical signals required for cell growth, tissue homeostasis, development and wound repair [55,69]. Our in vitro AEC wound repair data encouraged us to investigate the substrate roles of Fibronectin, Lumican and Periostin on AEC migration. The in vitro scratch wound repair system is not suitable to test the effectiveness of individual substrate components as the wounding process would disrupt the protein coating. We developed a novel ‘collagen drop’ cell migration assay to evaluate individual substrate component capacity to support or inhibit migration. Normal basement membrane architecture provides a favourable substrate for AEC migration whereas disrupted alveolar basement membrane and aberrant ECM remodeling play a crucial role in the abrogation of alveolar re-epithelialisation in pulmonary fibrosis [13]. Data from our ‘collagen drop’ cell migration assay has identified that both Fibronectin and Lumican are supportive of cell migration as substrate component. From consideration of the data obtained from wound repair and collagen drop assays we suggest a putative dual role (as topical and substrate components) of Fibronectin and Lumican, and a topical role of Periostin on alveolar epithelial cell migration and wound repair. Fibronectin, Lumican and Periostin have diverse biological effects on various cell types depending on their interactions with different receptor families, such as integrins, receptor tyrosine kinases (RTKs) and IGF-receptor [69-73]. It can be speculated that an individual candidate protein may stimulate different receptors depending on the mode of application with the potential to trigger different outcomes. Here our two different assay systems illustrated the effects of Periostin on AEC migration operated in an administration-dependent fashion. Further elucidation of hMSC secretory proteins and their interactions with target receptors in alveolar epithelial wound repair will provide a clear understanding in hMSC-mediated alveolar injury repair and regeneration.

## Conclusion

These data provide insight into potential hMSC paracrine influences on epithelial wound repair during alveolar injury repair. Aberrant alveolar re-epithelialisation is believed to be one of the major contributing factors for uncontrolled fibrogenesis in IPF [13]. Therefore, stimulation of re-epithelialisation within the alveolar regions could be a potential active vector for alleviation of the pro-fibrogenic processes as a curative therapeutic measure for IPF. Previous animal model-based studies demonstrated that MSC-paracrine factors attenuate pulmonary fibrosis through modulation of inflammation, suppression of fibrogenesis and stimulation of angiogenesis [19,20,31,33]. Here, we have demonstrated the efficacious influence of the hMSC secretome in the modulation of alveolar and small airway epithelial cell dynamics resulting in stimulation of wound repair. In-depth understanding of the humoral mechanisms of hMSCs in epithelial wound repair and regeneration will provide extended therapeutic options for clinical application of hMSC or their secretory products for fibrotic lung diseases, such as IPF.

## Competing interests

The authors declare that they have no competing interests.

## Authors’ contributions

Conceived and designed the study: KMA, MAS, NRF. Performed the experiments: KMA, SS. Analysed the data and prepared results: KMA. Wrote the manuscript: KMA, NRF; MAS. Study supervised: NRF, MAS. All authors read and approved the final manuscript.
